# Advancements of artificial intelligence in Chinese herbal medicine recommendation: A comprehensive review of data-driven approaches and clinical applications form 2016 to 2025

**DOI:** 10.1097/MD.0000000000048468

**Published:** 2026-05-01

**Authors:** Meng Yang, Jing Jin, Ya-Li Liu, Long Zhao, Hui Chen, Wei-An Hao, Xin-Yi Ao, Zhi-Lin Ran, Zhi Li, Xin Zhou

**Affiliations:** a The Affiliated Traditional Chinese Medicine Hospital, Southwest Medical University, Luzhou, China; b First Affiliated Hospital, Naval Medical University, Shanghai, China; c The Key Laboratory of Integrated Traditional Chinese and Western Medicine for Prevention and Treatment of Digestive System Diseases of Luzhou City, Affiliated Traditional Medicine Hospital of Southwest Medical University, Luzhou, China; d Institute of Integrated Traditional Chinese and Western Medicine, Southwest Medical University, Luzhou, China.

**Keywords:** clinical decision support, graph neural network, knowledge graph, syndrome differentiation, traditional Chinese medicine recommendation

## Abstract

Chinese herbal medicine recommendations are a core part of personalized traditional Chinese medicine (TCM) diagnosis and treatment. However, the complexity of the multidimensional relationships in syndrome differentiation and treatment, herbal compatibility, and dosage selection poses significant challenges to clinical decision-making. Although artificial intelligence technology has made remarkable progress in TCM auxiliary diagnosis, a systematic review of Chinese herbal medicine recommendation methods remains lacking. This review aims to address this gap by systematically reviewing Chinese herbal medicine generation methods grounded in knowledge graph-based recommendations, deep learning-based recommendations, and hybrid model-based recommendations from 2016 to 2025. Major TCM databases that serve as foundational data sources, including traditional Chinese medicine systems, pharmacology database, and analysis platform, symptom mapping database, High-throughput Experimental and Reference Database, and Traditional Chinese Medicine Information Database, which are crucial for training these recommendation models. It further analyses their evolutionary technical patterns and clinical applicability, providing critical references for developing theoretically robust and clinically interpretable artificial intelligence models for TCM practice. Existing research focuses on constructing knowledge graph-driven Chinese herbal medicine recommendation models, which enhance the interpretability of recommendations by structuring the relationships among symptoms, Chinese herbal medicines, and diseases. Meanwhile, a clinical data-driven framework is introduced to discover potential patterns from real-world diagnosis and treatment scenarios. Deep learning-driven methods are adopted to achieve end-to-end feature learning for TCM knowledge reasoning. To improve the clinical applicability of Chinese herbal medicine recommendation models, a few studies have reported evaluation methods by experienced clinical doctors using herbal effectiveness and herbal compatibility scores to assess the reliability of the models and the accuracy of the recommendation results. Forming a comprehensive evaluation system may be the development trend of the evaluation system for clinical decision-support systems. This review outlines a theory–data–clinical ternary evaluation framework for Chinese herbal medicine recommendation models, providing a methodological innovation for developing intelligent systems that meet the standards of evidence-based medicine.

## 1. Introduction

Traditional Chinese medicine (TCM) is a gem of Chinese civilization and an incredible creation of the Chinese nation. On May 25, 2019, the World Health Organization included TCM in the 11th International Classification of Diseases Revision. This classification came into effect in World Health Organization member states in 2022, marking the widespread recognition of TCM worldwide and bringing new opportunities for its global dissemination and development.^[1,2]^

The TCM diagnosis and treatment process is based on multidimensional associative reasoning.^[3]^ TCM encompasses a vast and unique knowledge system, integrating diagnosis, syndrome types, treatment methods, herbal medicines, and preventive healthcare (all intricately interconnected through specific nodes). When consulting a patient, a senior TCM physician first collects disease-related information via the 4 diagnostic methods (inspection, auscultation-olfaction, inquiry, and palpation) in accordance with fundamental TCM theories. Drawing on extensive clinical experience, the physician then performs in-depth analysis and reasoning on the complex clinical data to formulate an appropriate herbal prescription.

However, this traditional experience-based diagnosis and prescription reasoning approach has obvious limitations. On the one hand, it is highly subjective. The basic knowledge reserves and clinical experience levels of TCM physicians vary widely. Different TCM physicians may make different diagnoses and prescribe different prescriptions for the same patient, which, to some extent, affects the stability and reliability of clinical efficacy. On the other hand, it is inefficient. Due to the complexity of the TCM knowledge system and individual differences, TCM physicians need to spend a great deal of time and energy on diagnosis and prescription-writing. Especially when facing many patients, it is challenging to meet the demand for efficient diagnosis and treatment. Moreover, TCM prescriptions vary according to factors such as an individual’s age, gender, symptoms, and syndrome types, showing the characteristic of ``one syndrome, one prescription’’ (Yizheng Yifang). This makes it quite challenging to promote TCM prescription reasoning methods worldwide. Therefore, improving the interpretability of TCM prescription decision-making has become a key issue that TCM researchers focus on.

Artificial intelligence (AI) algorithms have demonstrated high efficiency in modeling complex relationships. They can rapidly process and analyze massive amounts of data, uncovering the hidden laws and patterns within the data to provide strong decision support, making.^[4,5]^ In recent years, with the continuous development of AI technology, its application in the field of TCM has become increasingly widespread.^[6]^ In TCM syndrome diagnosis, some researchers have developed a convolutional neural network (CNN) model based on deep learning. This model can accurately classify and diagnose patients’ syndromes by learning from many TCM clinical data.^[7,8]^ In predicting targets, graph convolutional network (GCN) and recurrent neural network have been applied.^[9]^ These models can extract features from multiple aspects of data, such as the chemical components and pharmacological effects of Chinese herbal medicines, to predict the targets and efficacy of Chinese herbal medication.^[10]^

Precise Chinese herbal medicine recommendations are crucial to clinical diagnosis and treatment in TCM. It helps TCM physicians improve the efficiency of clinical decision-making and reduce the influence of subjective factors. Although researchers have reported many excellent Chinese herbal medicine recommendation models, current relevant studies are scattered and lack systematic summarization and analysis.^[11]^ Different models vary in terms of data sources, algorithm selection, and evaluation indicators, which makes it difficult to compare these models. Therefore, a comprehensive review and analysis of Chinese herbal medicine recommendation models is necessary.

This study aims to comprehensively review research progress on Chinese herbal medicine recommendation models. From a data-driven perspective, it systematically summarizes Chinese herbal medicine recommendation methods based on knowledge graphs (KGs), deep learning, and hybrid models. It also details the technological evolution patterns of these methods and explores their clinical applicability and the challenges they face. This review provides references for developing AI models with strong theoretical guidance and clinical interpretability, and promotes the development and application of Chinese herbal medicine recommendation technology. The highlights and main contributions of this survey can be summarized as follows:

1. Reviewed data-driven Chinese herbal medicine recommendation via KGs, deep learning, and hybrid models.
2. Analyzed theoretical guidance and clinical applicability of existing TCM prescription models.
3. Discussed challenges and development directions of TCM prescription models.

## 2. Data collection

**Figure 1.**
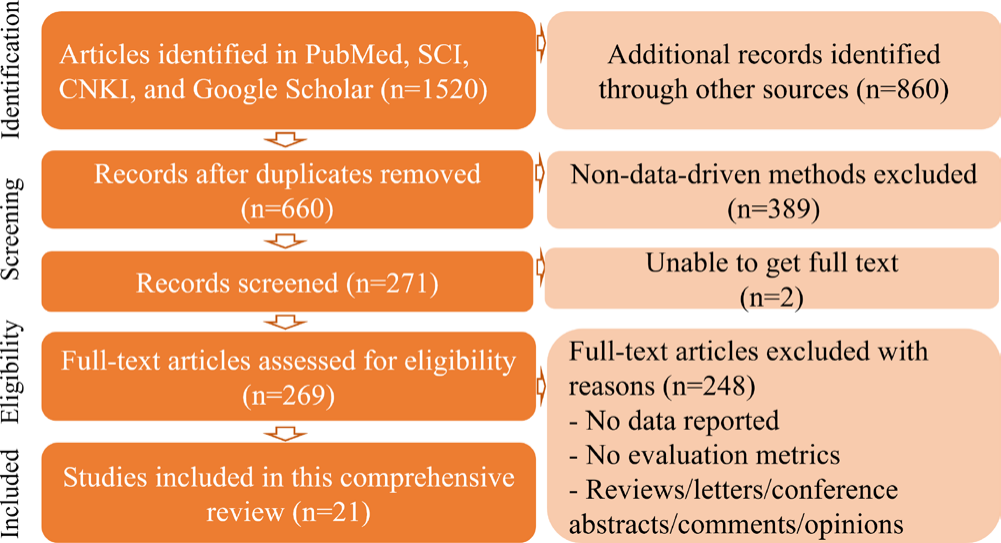
The process of data collection for this review.

In this study, a systematic search was conducted across major academic databases, including PubMed, Web of Science, and Google Scholar, with a time frame spanning from 2016 to 2025. The search keywords included: (“Traditional Chinese Medicine” OR “TCM”) AND (“herbal recommendation” OR “prescription generation”) AND (“knowledge graph” OR “deep learning” OR “AI”). Moreover, a manual search was carried out in conference paper repositories such as IEEE Xplore, arXiv, and ACL Anthology. Additionally, the reference lists of relevant reviews were traced back to ensure the inclusion of the latest research progress (Fig. 1).

*Inclusion criteria*: The research topics should focus on data-driven (KG, deep learning, hybrid models) Chinese herbal medicine recommendation methods, including clinical decision support systems and personalized prescription generation. Experimental or clinically-verified studies. The data processing involves structured KGs or deep learning models, and reproducible experimental results (e.g., accuracy, F1-score, human evaluation data) are provided.

*Exclusion criteria*: Case reports, reviews, and literature in languages other than Chinese and English. Non-data-driven methods without machine-learning procedures. Duplicated studies.

All the extracted data were cross-verified by 2 individuals. Two researchers independently entered information such as the technology type, dataset type, data size, and evaluation process. Any discrepancies were reexamined against the original texts.

## 3. Multidimensional classification of TCM data

**Figure 2.**
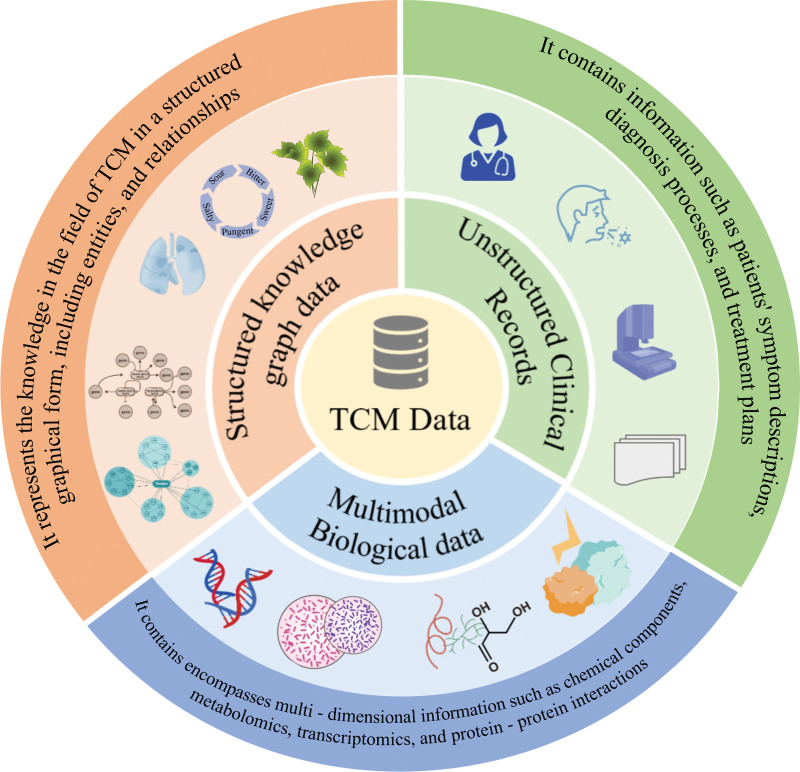
Multidimensional classification of TCM data. Structured KG data is systematically organized, graphically presented, and well-structured for efficient computer storage, query, and reasoning. Unstructured clinical records in text and image forms need natural-language-processing technologies for information extraction. Multimodal biological data, from diverse detection techs and devices, reflects organisms’ physiological and pathological states from multiple perspectives. KG = knowledge graph, TCM = traditional Chinese medicine.

TCM data serves as the cornerstone for AI model development, and its complexity is closely related to the holistic nature of TCM theory. Based on their sources and structures, TCM data can be classified into structured KG data, unstructured clinical records, and multimodal biological data (Fig. 2).

### 3.1. Structured KG data

Structured KG data represents the knowledge in the field of TCM in a structured graphical form. It includes entities (such as Chinese herbal medicines, prescriptions, and diseases), attributes (such as the nature, flavor, meridian tropism, and toxicity of Chinese herbal medicines), and relationships (such as the composition relationship between Chinese herbal medicines and prescriptions, the treatment relationship between diseases and Chinese herbal medicines, and the attribute relationship between dosage and toxicity).^[12]^ In TCM KGs, triples are widely used to express various relationships.^[13]^ For example, Ephedra (Ma Huang)–Efficacy–Inducing sweating and relieving exterior syndromes indicates that Ephedra, a Chinese herbal medicine, is effective in inducing sweating and relieving exterior syndromes. Meanwhile, the graph structure can intuitively display the associations between TCM entities.^[14,15]^ The TCM meridian system is the pathway for the circulation of qi and blood in the human body. If acupoints are regarded as nodes and meridians as edges, on the Bladder Meridian of Foot (Taiyang, the Jingming Point and the Cuanzhu Point are connected by a meridian, which is represented as 2 nodes connected by an edge in the graph). TCM researchers can use Protégé to construct the ontology of TCM KGs, which facilitates the definition of concepts and relationships and provides a semantic framework for constructing TCM KGs.^[16–18]^ Neo4j is widely used to store and manage large-scale TCM research data, where patient information, disease diagnoses, used prescriptions, and Chinese herbal medicines are all regarded as nodes, and the relationships between them are considered edges.^[19–21]^ Therefore, the KG can intuitively present the associated relationships among structured data such as Chinese herbal medicines, prescriptions, diseases, and symptoms.

Some large-scale KGs in the field of TCM will record in detail information such as the name, origin, nature, flavor, meridian tropism, and main-treated diseases of each Chinese herbal medicine, as well as the dosage of each Chinese herbal medicine in prescriptions and the compatibility relationships. The TCM Knowledge Service Platform (http://www.tcmkb.cn) covers 2.1 million data resources, including ontologies, terminologies, literatures, and knowledge bases in the TCM field, presenting TCM knowledge intuitively as a graph. Traditional Chinese medicine Systems Pharmacology Database and Analysis Platform (https://www.tcmsp-e.com) covers Chinese herbal medicines (499 kinds), chemical components (29,384 kinds), target proteins (3311), and disease-related data. It focuses on systematic pharmacological analysis and supports the construction and visualization of drug–component–target–disease networks, which are used to screening of active components of Chinese herbal medicines, the study of multi-target action mechanisms, and the prediction of compound efficacy.^[22]^ Traditional Chinese medicine information database (https://www.bidd.group/TCMID) provides the composition, dosage, indications, and modern pharmacological research results of TCM formulas.^[23,24]^ symptom mapping database (http://www.symmap.org/) encompasses 1717 TCM symptoms, correlating them with 499 herbs used in modern medicine and 961 symptoms. Meanwhile, researchers have collected 5235 diseases associated with these symptoms, 19,595 herbal components, and 4302 target genes, constructing a large-scale heterogeneous network that includes all these elements.^[25]^ Symptom mapping database integrates TCM and modern medicine in common aspects at the phenotypic and molecular levels. High-throughput Experimental and Reference Database (http://herb.ac.cn/) integrates in vitro experimental data (such as activity screening and toxicity testing) and literature evidence of TCM components, and provides the binding affinity (Kd, IC50) between components and targets and details of experimental methods.^[26,27]^ This structured KG data facilitates the retrieval and reasoning of TCM knowledge.^[28]^

### 3.2. Unstructured clinical records

Unstructured clinical records mainly come from medical records, doctors’ orders, and progress notes in TCM clinical practice. They exist in natural language text and contain information such as patients’ symptom descriptions, diagnosis processes, and treatment plans. Computer tools can be used for preprocessing to facilitate deep learning models to learn from this data. The bidirectional encoder representations from transformer (BERT)-based entity recognition model performs excellently in natural language processing tasks. After fine-tuning, TCM-related entities from unstructured text can be identified.^[29–32]^ The word vector tool Word2Vec converts words in text into vector representations to capture semantic similarities between words, enabling deep learning models to process text data.^[33–35]^ For example, Ginseng (Ren Shen) and Codonopsis (Dang Shen) have semantic similarities. After word vector conversion, their distances in the vector space will be relatively close, which helps the model learn this semantic relationship. Term frequency-inverse document frequency (TF-IDF) is used to evaluate the importance of a word in a document.^[36–38]^ In TCM clinical records, TF-IDF can help extract representative keywords as features for deep learning models. The TF-IDF values of some common general words (such as patient and symptom) are relatively low. In contrast, those of some specific TCM terms (such as Tianma Gouteng Decoction) are relatively high, which can better represent the core content of the document. Large language models, after being trained on a large amount of text data, can understand the complex language expressions and professional terms in TCM clinical records.^[39–43]^ Large language models use built-in algorithms to automatically identify and remove special characters, garbled codes, and irrelevant HyperText Markup Language tags in clinical records, greatly improving the efficiency of information extraction and analysis and saving human and time costs.

### 3.3. Multimodal biological data

Multimodal biological data in TCM encompasses multidimensional information such as chemical components, metabolomics, transcriptomics, and protein–protein interactions. Its significance lies in systematically analyzing TCM formulas’ complex mechanism of action. Different computer tools are needed to preprocess, extract features, and fuse this data to enable deep learning models to learn multimodal biological data effectively. The open-source tool PubChem (https://pubchem.ncbi.nlm.nih.gov/) contains much chemical substance information.^[44–46]^ It can query detailed information about specific chemical components to assist in data annotation and feature understanding. The web-based metabolomics data analysis platform MetaboAnalyst (https://www.metaboanalyst.ca/) provides rich data analysis and visualization functions to conduct multivariate statistical analysis of TCM metabolomic markers and mine potential biological information.^[47,48]^ Furthermore, the deep learning frameworks TensorFlow and PyTorch can fuse the processed chemical component data, metabolomic marker data, and transcriptomic data. The models can learn the association and complementary information among multimodal biological data in TCM by constructing appropriate network structures.^[49–51]^

Through the collaborative application of the above tools and methods, the utilization efficiency of structured and unstructured data in TCM can be significantly improved, laying a foundation for developing intelligent diagnosis and treatment systems.

## 4. Deep learning approaches for feature extraction from TCM data

Unlike Western medicine data, TCM encompasses vast amounts of unstructured textual information, including ancient classics, clinical records, and diagnostic notes. Automatically extracting entities (such as symptoms, syndromes, herbs, and formulas) and their semantic relationships from these texts is a critical step toward enabling intelligent recommendation systems that support clinical decision-making and multi-pharmacological research. Recent advances have explored feature extraction methods tailored for TCM texts from perspectives including network mining, sequence labeling, and pretrained models.

From a network mining perspective, researchers have constructed heterogeneous entity networks based on TCM literature corpora, where nodes represent TCM entities and edges denote candidate relationships. By introducing a Heterogeneous Factor Graph Model, the existence probabilities of all edges can be inferred simultaneously, with model parameters estimated using semi-supervised learning algorithms to effectively mine latent associations between entities.^[52]^ Similarly, Chen et al applied Heterogeneous Information Networks to the *Pharmacopoeia of the People’s Republic of China* corpus, modeling prescription entities and their attributes to achieve efficient classification of TCM formulas, offering a novel approach for structuring prescription knowledge.^[53]^

To address challenges such as ambiguous entity boundaries and terminological diversity in TCM clinical texts, researchers have developed specialized Named Entity Recognition methods. These approaches first leverage pretrained character embeddings from Wikipedia and TCM-relevant corpora to construct input representations. A set of TCM clinical term features is then introduced and integrated with pretrained vectors by incorporating a specialized length dimension. Finally, semi-supervised learning strategies are employed to train the model, significantly reducing manual annotation effort while maintaining recognition accuracy.^[54]^ This method effectively identifies entities such as symptoms, syndromes, and herbs, laying the foundation for subsequent relation extraction.

In recent years, pretrained language models exemplified by BERT have demonstrated remarkable capability in processing TCM texts. TCM clinical records contain extensive domain-specific terminology and context-dependent semantic information. By fine-tuning BERT on unlabeled clinical corpora, the model can achieve deep understanding of TCM linguistic characteristics, subsequently enabling text classification or sequence labeling tasks.^[55]^ This approach operates directly on Chinese character sequences without requiring complex preprocessing or feature engineering, substantially improving both the efficiency and accuracy of feature extraction from TCM texts.

## 5. Data-driven Chinese herbal medicine recommendation method

**Figure 3.**
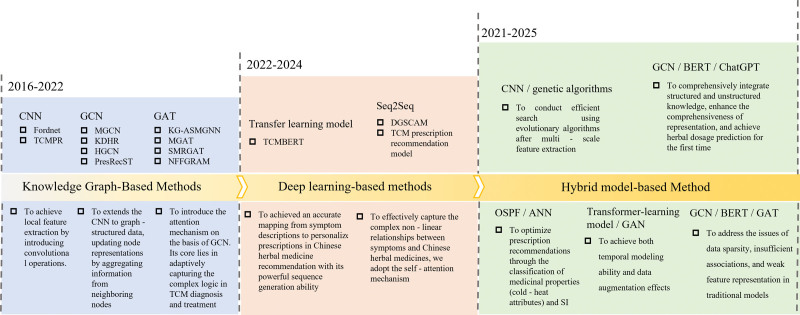
The advancement of recommendation models for Chinese herbal medicines. From 2021 to 2025, these models trended towards hybrids. The KG method builds a knowledge network of herbs, diseases, and efficacy for precise knowledge extraction and representation. Deep learning processes vast data, automatically learning complex features. The hybrid model overcomes single-method limitations, integrating knowledge- and data-driven advantages. KG = knowledge graph.

The core principle of Chinese herbal medicine recommendation lies in integrating the traditional syndrome differentiation theory and treatment with modern data science. Traditional TCM relies on compatibility principles and the theory of medicinal properties. At the same time, data-driven methods construct a multidimensional association network of symptoms, syndromes, Chinese herbal medicines, and targets by analyzing a vast amount of ancient medical literature, clinical records, and molecular pharmacology data, quantifying and generalizing empirical knowledge.^[56–58]^ This review is carried out from 3 levels (Fig. 3): KGs, deep learning, and hybrid models, systematically revealing the technological evolution patterns of Chinese herbal medicine recommendation.

### 5.1. KG-based methods

Artificial neural networks (ANNs) are crucial in integrating TCM empirical knowledge. They can extract semantic features from unstructured text and predict the potential efficacy of Chinese herbal medicine combinations.^[59–61]^ The knowledge-graph-based approach further enhances the interpretability and clinical adaptability of Chinese herbal medicine recommendation models.^[62,63]^ By constructing a multilayer KG of ``Chinese herbal medicines–components–targets–diseases,’’ the model can restore the logical core of TCM theory. CNNs, GCNs, and graph attention networks (GATs) have been used to handle various TCM machine-learning tasks.

A deep neural network, with its multilayer nonlinear hidden layer structure, can learn high-order abstract features from large-scale complex data. Theoretically, it can approximate any complex function, providing a fundamental framework for processing multisource heterogeneous data in TCM.^[64,65]^ On this basis, a CNN achieves local feature extraction by introducing convolutional operations. Its core architecture (convolutional layers, pooling layers, and fully connected layers) significantly reduces computational complexity through a parameter-sharing mechanism, while endowing the model with the advantage of translational invariance.^[66–69]^ This characteristic makes it uniquely valuable in TCM data analysis.

The data shows significant multimodal and multilevel complexity in the Chinese herbal medicine recommendation scenario. On the one hand, the symptom descriptions in electronic medical records (such as red tongue with yellow and greasy coating and stringy and slippery pulse) contain combinatorial semantic features, and local word-order associations must be captured. On the other hand, the compatibility rules of formulas imply a hierarchical network relationship of monarch, minister, assistant, and guide (MMAG) (Jun-Chen-Zuo-Shi). The CNN conducts a sliding-window analysis on symptom texts through multi-scale convolution kernels, which can accurately identify the local patterns of key symptom combinations. Take the formula recommendation via the deep neural network model as an example. This model extracts diagnostic features from more than 20,000 electronic medical records of the famous veteran TCM doctor Li Jiren, and combines the graph embedding technology of the herb–compound–target network to achieve the fusion of phenotype–molecule phenotype–molecule bimodal data innovatively.^[70]^ Experiments show that introducing molecular information increases the Hit ratio@10 of Chinese herbal medicine recommendation by 46.89% compared with the best baseline method. This random forest verifies the potential of CNN in cross-modal knowledge integration.

At the technical implementation level, CNN-based Chinese herbal medicine recommendation endeavors to overcome 2 core challenges. On the one hand, it is about standardizing unstructured data. Free-text medical records must be converted into structured features through natural language processing. The subnetwork-based symptom term mapping method developed by the traditional Chinese medicine prescription recommendation (TCMPR) model dynamically extracts local subnetworks (such as the cluster of associated symptoms centered around headache) from the global KG, and maps unrecorded symptoms (e.g., head distending pain) to subgraph structures with similar semantics, effectively addressing the issue of term fragmentation.^[71]^ On the other hand, it involves the quantitative embedding of TCM theory. The formula recommendation via deep neural network combines network pharmacology with CNN and encodes the rules of properties, tastes, and meridian tropism through the embedded representation of the herb–target network.^[70]^ The TCMPR further introduces metapath modeling, defines the high-order relationship chain of symptoms–syndromes–treatment methods–prescriptions, and mines the logical path of syndrome differentiation and treatment from the Chinese herbal medicines-symptom-related KG, achieving a precision of 28.23% in the recommendation results.^[71]^ Therefore, CNN can handle the surface features of TCM data and deeply integrate into the core of TCM theory through architectural improvements, promoting intelligent diagnosis and treatment evolution from static rule matching to dynamic semantic reasoning.

The GCN extends the CNN to graph-structured data, updating node representations by aggregating information from neighboring nodes. The ability of GCN to aggregate information between nodes and handle complex graph-structured data shows a unique value in the intelligent research of TCM.^[72–74]^ Its core advantage lies in capturing the multidimensional dynamic associations in TCM diagnosis and treatment through the message-passing mechanism. In short, it conducts deep learning on the intricate n-ary associations among symptoms, state elements, syndromes, and herbs. The multigraph convolutional network (MGCN) model constructs 2 subgraphs: the symptom-state element-symptom (Se graph) and the symptom-syndrome-symptom (Ts graph), which simulate the logical chain of TCM syndrome differentiation and significantly improve the accuracy of Chinese herbal medicine recommendation.^[75]^ This ability is reflected in the modeling of static relationships and extends to dynamic clinical scenarios. The TCM prescription recommendation network architecture for real-world patients with integration of syndrome differentiation and treatment planning model uses a self-built TCM Lung disease knowledge graph, which contains 14,948 samples, 1801 symptoms, 54 syndromes, 67 treatment methods, and 410 herbs.^[76]^ The TCM-Lung integrates symptom classification, treatment method determination, and Chinese herbal medicine recommendation into a progressive graph network. Its phased reasoning process highly conforms to the TCM doctors’ diagnosis and treatment process. It combines the 4 diagnostic methods-syndrome differentiation, treatment principles, prescribing prescriptions, and demonstrating a closed-loop adaptability from theory to clinical practice.

To overcome the limitation of semantic sparsity, the knowledge-driven herb recommendation (KDHR) model constructs a KG that includes attributes such as the 4 natures and 5 tastes and meridian tropism of TCM. It encodes TCM theories like *Scutellaria baicalensis* (Huang Qin), which is bitter and cold and enters the lung meridian, and *Coptis chinensis* (Huang Lian), which clears heat and dries dampness into structured node features. Jointly training with the symptom–herb association data in historical prescriptions, it breaks through the limitation of traditional bag-of-words models in perceiving complex semantics.^[77]^ Through the attention mechanism to dynamically weight key paths, KDHR aggregates the directly associated syndromes of symptoms (e.g., fever and aversion to cold are mapped to Taiyang disease), and integrates herb attributes and prescription co-occurrence patterns across layers. For example, the model represents the classic syndrome-differentiation logic of Taiyang disease-Guizhi Decoction as a high-weight metapath. Yang et al introduced the heterogeneous graph neural network into the field of TCM formula recommendation.^[78]^ Their research is based on 480 Chinese herbal medicines, 12,735 compounds, and 24 SARS-CoV-2 proteins. They constructed an heterogeneous graph neural network of herb–compound–protein through virtual screening. This network covers molecular docking data and embeds the semantics of TCM theory through metapaths. For instance, the herb–compound–structural protein path simulates the mechanism of action of the property of clearing heat and removing toxins, while the herb–compound–nonstructural protein path corresponds to the treatment principle of strengthening the healthy qi and eliminating the pathogenic factors. At the same time, the Variational Graph Auto-Encoder is used to capture the evolution law of the COVID-19 course (mainly characterized by fever and cough in the early stage and accompanied by pulmonary fibrosis in the later stage), and then realize the phased recommendation of compound prescriptions. These studies together promote the Chinese herbal medicine recommendation to move from static rule matching to a dynamic mechanism-driven intelligent diagnosis and treatment paradigm.

The GAT introduces the attention mechanism based on GCN. By dynamically allocating the relationship weights between nodes and fusing multisource data, GAT has opened up a new path with accuracy and interpretability for Chinese herbal medicine recommendation.^[79–82]^ Its core lies in adaptively capturing the complex logic in TCM diagnosis and treatment. The meta-path guided graph attention network model innovatively constructs an integrated KG, achieving a deep alignment between TCM theory and modern medical pharmacological analysis.^[83]^ Meta-path guided graph attention network guides information propagation through predefined meta-paths, allowing it to more specifically explore the potential relationships in TCM data. Moreover, it selects long-range path instances with high attention weights to generate fine-grained explanations. In Chinese herbal medicine recommendations, the model can provide recommended prescriptions and elaborate on why these herbs are selected and how they interact synergistically. The syndrome-aware KG-enhanced attentive multigraph neural network (KG-ASMGNN) further utilizes the attention mechanism to optimize the symptom-herb mapping. Combining KG features with a multilayer perceptron improves the F1-score in the fine-grained syndrome differentiation task, especially enhancing the generalization ability for rare syndromes (such as true cold with false heat).^[84]^ To fully learn the interaction between symptoms and herbs, the MultiGraph Residual Attention Network and Semantic Knowledge Fusion model uses the natural language processing model Word2vec to integrate the identity information of TCM symptoms themselves and learn the contextual semantic features of symptom entities.^[85]^ At the same time, it incorporates the quantified herb attributes as semantic enhancement features into the model. This method breaks through the limitation of traditional models that only rely on single data features, and can more comprehensively capture the semantic associations between symptoms and herbs. The MultiGraph Residual Attention Network and Semantic Knowledge Fusion constructs symptom–symptom, symptom–herb, and herb–herb graphs, respectively, and uses residual connections to fuse interaction features, capturing the relationships between symptoms and herbs from different perspectives. The nonlinear multifeature fusion and gated recurrent self-attention mechanism introduces an enhanced bipartite graph diffusion algorithm and combines it with a gated recurrent self-attention mechanism to predict the associations between herbs and symptoms.^[86]^ The introduction of the attention mechanism reflects the transformation of Chinese herbal medicine recommendation from a purely data-driven recommendation mode to a precise one driven by theoretical knowledge and action mechanisms (Table 1).

**Table 1:** Comparison of neural network models.

Model	Advantages	Limitations	References
ANN	Simple to implement, suitable for basic tasks	Limited expressive power, cannot handle complex nonlinear relationships	Tang et al^[59,60]^; Liu and Du^[61]^
DNN	Multilayer structure for high-dimensional feature extraction, versatile	Requires large datasets, inefficient for unstructured data	Xu et al^[65]^; Wei et al^[64]^
CNN	Efficient local spatial feature extraction, parameter sharing, reduces computational costs	Limited to grid-like data, cannot directly process graph structures	Sun and Qian^[67]^; Jiang et al^[66]^; Bajić et al^[69]^; Luo et al^[68]^
GCN	Directly processes graph data, captures node dependencies	Assumes equal importance among neighboring nodes, lacks flexibility	Dai et al^[72]^; Chen et al^[74]^; Zhou et al^[73]^
GAT	Dynamically learns neighbor weights, ideal for heterogeneous graphs and key relationship mining	High computational complexity, sensitive to noise	Dai et al^[81]^; Jiang et al^[80]^; Li et al^[79]^; Zhang et al^[82]^

ANN = artificial neural network, CNN = convolutional neural network, DNN = deep neural network, GAT = graph attention network, GCN = graph convolutional network.

### 5.2. Deep learning-based methods

Deep learning provides powerful technical support for Chinese herbal medicine recommendation through multi-level feature abstraction and nonlinear relationship modeling. Its core advantage lies in the ability to automatically extract high-order associated features from massive heterogeneous data (such as symptom descriptions, tongue image data, and molecular targets), which helps to deeply structure and dynamically adapt the TCM knowledge system.^[87–90]^

The Seq2Seq model, with its powerful sequence generation ability, has achieved an accurate mapping from symptom descriptions to personalized prescriptions in Chinese herbal medicine recommendation, serving as a crucial bridge connecting traditional experience and modern computing. The core advantage of this model lies in its flexibility in handling variable-length inputs and outputs. In TCM diagnosis and treatment, patients’ symptoms often present in complex combinations (e.g., aversion to cold and fever, no sweating, floating and tense pulse), and prescriptions need to dynamically match the compatibility of multiple herbs (for example, Mahuang Decoction contains 4 herbs). In short, treatments are tailored to individual symptoms. The encoder-decoder architecture of Seq2Seq is naturally suitable for such tasks. Ren et al used a bidirectional GRU to encode symptom texts. They combined it with the pretrained embedding representations from a KG (such as the complex [space vectors of fever] Taiyang disease-Cinnamon Twig [Gui Zhi]).^[91]^ The decoder then gradually generates herb sequences. A multi-hot vector was introduced to avoid repeated recommendations, reducing the repetition rate of prescriptions. The dual-branch guidance strategy, combined with the candidate attention model (DGSCAM), innovatively designed a dual-branch guiding strategy: one branch analyses the semantics of symptoms (e.g., cough with yellow phlegm corresponds to lung heat), and the other branch constructs a candidate herb pool using historical prescriptions. Through the attention mechanism, compliant combinations are dynamically selected (e.g., Scutellaria baicalensis-Fritillaria [Bei Mu] thunbergii enhances the efficacy of clearing heat and resolving phlegm), thereby improving the clinical relevance of the generated prescriptions.^[92]^

At the technical implementation level, deep learning methods promote the in-depth integration of the TCM knowledge system and the prescription generation model. The KG completion technology encodes theories such as properties, flavors, and meridian tropism and the taboos of 18 incompatible medicaments and 19 medicaments of mutual restraint into computable embedding vectors, enabling the model to understand the deep-seated associations like Ephedra-inducing sweating to release the exterior.^[93,94]^ The DGSCAM develops a task-qualified knowledge base matching module, transforming the MMAG rules into attention weight constraints.^[92]^ In a TCM formula, the weight of the sovereign herb (such as Ephedra) is over 0.75, while the assistant and guide herbs (such as Licorice Root [Gan Cao]) undertake the function of harmonizing. At the same time, contraindicated combinations (such as Aconite [Wu Tou]–Pinellia Tuber [Ban Xia]) are filtered in real-time to ensure the safety of prescriptions. This knowledge injection strategy compels the generated results with classical TCM theories.

Applying the transformer model to Chinese herbal medicine recommendations demonstrates multiple advantages. Its core lies in the self-attention mechanism, which can effectively capture the complex nonlinear relationships between symptoms and Chinese herbal medicines. Take TCMBERT as an example.^[95]^ First, the self-attention mechanism can globally capture the complex associations among symptoms, medical histories, and Chinese herbal medicines. For instance, in the syndrome type of stagnation of liver qi and deficiency of spleen, the model can simultaneously analyze the compatibility logic between main symptoms such as abdominal distension and fatigue and herbs like Bupleurum Root (Chai Hu) and Largehead Atractylodes (Bai Zhu) Rhizome. Secondly, the transfer learning framework breaks through the data bottleneck through a two-stage training strategy. In the pretraining stage, the model learns the semantic rules of over 450,000 sentence pairs from ancient medical books such as “Treatise on Febrile Diseases” and “Medical Records of Liu Bichen,” enabling it to master prior knowledge of theories such as the 4 natures and 5 flavors and meridian tropism. In the fine-tuning stage, only 517 clinical cases are needed to generate prescriptions that conform to TCM diagnosis and treatment logic, overcoming the limitation that traditional models require >90% of labeled data. Notably, the triple-attention mechanism automatically weights core symptoms (e.g., fever without sweating) at the symptom attention layer, integrates patients’ physical characteristics (e.g., deep and thready pulse) at the medical history attention layer, and dynamically adjusts the compatibility strategy during the generation process (for example, the pungent [warm synergistic effect of Ephedra] Cinnamon Twig is higher than that of Ephedra-Gypsum [Shi Gao], to reduce the generation probability of Gypsum).

Furthermore, to identify precise therapeutic targets for Alzheimer disease, Chen et al employed support vector machine and multiple linear regression to develop predictive models. Notably, they also applied deep learning methods and random forest algorithms to identify compounds capable of forming stable interactions with glycogen synthase kinase-3β, specifically methyl 3-O-feruloylquinate from *Phellodendron amurense* and cynatratumin A from *Cynanchum atratum*. Unfortunately, these findings have not been validated experimentally or clinically.^[87]^

### 5.3. Hybrid model-based method

The application of the hybrid model in Chinese herbal medicine recommendation has significantly improved the accuracy and safety of prescriptions through the collaboration of multiple technologies. TCMRF (GCN integrated with BERT and further integrated with Chatgpt) deeply integrates the molecular KG with the semantics of ancient TCM books.^[96]^ The GCN analyses the inhibitory effect of active ingredients in Chinese herbal medicines on specific signaling pathways and quantifies the targeting efficacy of herbs. The BERT model extracts the semantic associations of promoting blood circulation and removing blood stasis from the text of *Synopsis of Prescriptions of the Golden Chamber* and identifies the compatibility rules of the classic herb pair Salvia Miltiorrhiza (Dan Shen)–Ligusticum Wallichii (Chuan Xiong). Chatgpt enhances the contextual understanding of vague symptoms through generative dialogue. This cross-modal integration ensures that the generated prescriptions not only comply with traditional prescription rules but also dynamically match Chinese herbal medicines with the main symptoms of patients through the semantic model. Finally, the multi-task loss function is combined to predict the dosage of Chinese herbal medicines simultaneously.

In terms of dynamic adaptation, the transformer combined with a generative adversarial network model demonstrates the dual advantages of sequential modeling and data augmentation. Zhang et al used sequential encoding of 21,295 electronic medical records.^[97]^ The model captured the evolution of the pathological mechanism of patients from white and greasy tongue coating to yellow and dry tongue coating and dynamically adjusted the prescription strategy. In the early stages, it is recommended that Huoxiang Zhengqi San be used to resolve dampness and relieve the exterior. In the later stages, it was switched to Baihu Tang to clear heat and promote fluid production. Meanwhile, the noisy augmented samples generated by generative adversarial network (by adding random pulse wave data) effectively expanded the diversity of the training set. This enabled the model to maintain a high recall rate when dealing with rare syndrome types (such as the complex pattern of cold and heat in COVID-19), overcoming the traditional methods’ reliance on balanced datasets.

The safety assessment achieved an innovative breakthrough through an ontology-based side-effect prediction framework and ANN.^[98]^ This model is based on the ontology of cold and heat properties. It classifies the components of COVID-19 prescriptions into hot-natured (e.g., Aconiti Lateralis Radix Praeparata [Fu Zi]) and cold-natured (e.g., Gypsum Fibrosum), constructs prescription vectors, and inputs them into an ANN to calculate the safety index (SI). Among them, Maxing Shigan Tang obtained an SI of 0.85 due to the balanced cold-heat compatibility of Gypsum Fibrosum (Ephedrae Herba, while the combination of Aconiti Lateralis Radix Praeparata) Trichosanthis Fructus (Gua Lou) was automatically excluded because it violated the 18 incompatibilities taboo. This medicinal property constraint mechanism reduced the incidence of clinical side-effects of 10 highly safe compound prescriptions (such as Qingfei Paidu Decoction) in the recommended prescriptions. At the same time, through dynamic weight adjustment, Jinhua Qinggan Granules with SI > 0.8 were preferentially recommended in the early stage of the epidemic, taking into account both emergency treatment and medication safety.

For complex diseases such as liver cancer, combining multi-scale CNN and genetic algorithm achieved multi-scale feature extraction and evolutionary optimization.^[99]^ A multi-scale CNN hierarchically extracted features from molecular structures (e.g., the hydroxyl groups on quercetin’s benzene ring), target networks (the IL-1β core module), and The Cancer Genome Atlas clinical data, identifying the dual-pathway inhibitory effect of the *Scutellaria barbata* (Ban Zhilian)–*Hedyotis diffusa* (Baihua Sheshe Cao) combination on the MMP9 protein. A genetic algorithm simulated natural selection, screening for the prescription with the highest target enrichment through crossover and mutation over tens of thousands of iterations (e.g., replacing Sparganii Rhizoma [E Zhu] with Curcumae Zedoariae Rhizoma [San Leng]), which significantly improved training set accuracy.

The Chinese herbal medicine recommendation based on semantically enhanced self-supervised graph convolution and multi-head attention fusion model introduces a residual GCN to address the issues of insufficient correlation between prescriptions and data sparsity.^[100]^ Modeling the complex topological relationships among prescriptions, symptoms, and herbs enhances the ability to represent structured knowledge. To further optimize feature representation, the model combines a self-supervised learning mechanism. It generates supervision signals using unlabeled data, reducing the dependence on labeled data and improving generalization ability. Finally, semantic features, graph structure information, and herb attributes are dynamically fused through the multi-head attention mechanism to achieve the interaction of multidimensional features and weight assignment. This enhances the accuracy of recommendations and improves the interpretability of the results. This design strategy that integrates natural language processing, GNN, self-supervised learning, and attention mechanisms demonstrates the technical synergy advantages of the hybrid model and provides an innovative solution for intelligent recommendation in the field of TCM.

Additionally, network analysis and collaborative filtering algorithms have been applied to Chinese herbal medicine recommendation. Network analysis constructs a “target–molecule–herb” multilayer network to elucidate complex herb–disease interactions. In Alzheimer disease research, the traditional Chinese medicine formula prediction model leverages protein–protein interaction networks to identify regulatory relationships between *Polygonum multiflorum* components and Aβ protein. Combined with a random walk algorithm, it quantifies network influence, offering molecular insights into the traditional theory of “tonifying the kidney and replenishing the marrow.”^[101]^ The collaborative filtering algorithm mines implicit compatibility rules from historical data. Its core is constructing a Chinese herbal medicine efficacy matrix and predicting potential combinations through Laplace matrix decomposition. In the research on COVID-19, Yao et al analyzed the high-frequency herb pairs (such as Ephedrae Herba-Armeniacae Semen [Xing Ren]) in the ancient prescription database and combined the molecular docking data (binding energy calculated by q-vina) to screen out combinations with synergistic antiviral effects, such as Lonicerae Japonicae Flos (Jin Yinhua)–Forsythiae Fructus (Lian Qiao).^[102]^ This method, which integrates modern pharmacology and traditional experience, effectively alleviates the cold-start problem caused by the lack of historical prescriptions for newly emerging diseases.

The above-mentioned basic models have different focuses when performing the Chinese herbal medicine recommendation task, mainly due to the various requirements for modeling specific TCM data and knowledge fusion. Table 2 analyses the performance of representative models.

**Table 2:** Details of representative TCM recommendation models.

Technology type	Model name	Year	Dataset type	Data size	Data preprocessing tool	*Precision*	*Recall*	*F1 score*	*Hit ratio@10*	Human qualitative evaluation metrics	Innovation points	References
CNN	Fordnet	2021	Clinical data	6393 EHRs from Yijishan hospital	Artificial Neural Network	-	-	-	46.89	-	To combine phenotypic and molecular information, use CNN to extract features of diagnostic descriptions, and adopt a data augmentation strategy	[70]
	TCMPR	2022	KG + Clinical data	15,845 pieces of consultation information from clinical case data	Artificial Neural Network	28.23	12.98	17.78	-	-	To solve the representation problem of unrecorded symptoms through SSTM, a CNN for feature extraction is used.	[71]
GCN	MGCN	2022	KG	1329 symptoms, 1641 state-elements, and 358 syndrome-types from *the Treatise on Febrile Diseases dataset*	Heterogeneous graph	62.70	73.39	67.52	-	-	To emphasise capturing the “n-ary relationships” in TCM diagnosis	[75]
	KDHR	2022	KG	33,765 pieces of data contain 390 symptoms and 805 herbs from published research^[103]^	Heterogeneous graph	21.38	15.10	17.70	-	-	To enhance the perception of complex associations between symptoms and herbs by constructing a herbal KG and introducing herbal property information	[77]
	HGCN	2022	KG	480 herbal medicines, 12735 associated chemical compounds and 24 SARS-Cov-2 proteins from TCMSP database	Heterogeneous graph	-	-	-	-	-	To construct a multi-layer network of “herbs-compounds-viral proteins” and implement a precise treatment strategy from molecular targeting to the staging of the COVID-19 disease course	[78]
	PresRecST	2024	Clinical data	14948 samples, 1801 symptoms, 54 syndromes, 67 treatment methods, 410 herbs from the Department of Respiratory Medicine, The Frist Affiliated Hospital of Henan University of Chinese Medicine	Heterogeneous graph	60.25	17.75	27.43	-	-	To integrate symptom collection, syndrome differentiation, treatment method determination and herbal recommendation, emphasising the coherence of the clinical process	[76]
GAT	MGAT	2023	KG	26,360 prescriptions, 360 symptoms, and 753 herbs^[104,105]^	Heterogeneous graph	29.41	21.05	-	-	-	To emphasise guiding information propagation through predefined meta-paths and selecting paths with high attention weights to generate explanations	[83]
	KG-ASMGNN	2021	KG	26360 prescriptions, 360 symptoms, 753 herbs, and 947 lung cancer prescriptions^[103,104]^	Heterogeneous graph	29.11	23.34	16.92	-	-	To use the attention mechanism and MLP to refine the symptom-herb mapping and combine the KG node features with multi-graph propagation to enhance representation learning	[84]
	SMRGAT	2023	KG	9196 prescriptions, 1448 symptoms, and 1919 herbs from ETMC database and yaozh database	Heterogeneous graph	15.11	20.60	18.25	-	-	To utilise Word2vec to extract the semantic features of symptoms and combine them with herb attributes to construct a multi-graph attention model	[85]
	NFFGRAM	2025	Public database	26360 prescriptions, containing 360 symptoms and 753 herbs^[106,107]^	Heterogeneous graph	21.77	12.46	19.28	-	-	The herb-symptom association matrix is reconstructed by initiating the process with the fractal-weighted K nearest neighbour algorithm.	[86]
SVM + MLR	TCM formula prediction for Alzheimer’s Disease	2019	Public database	18776 compounds	-	-	-	-	-	-	To investigate the TCM candidates that can dockwell in GSK3β for the treatment of Alzheimer ‘s disease	[87]
Seq2Seq	TCM prescription recommendation model	2024	KG	32,937 prescriptions, 1,820 distinct symptoms, and 1,682 types of medicinal materials from the TCM modernization database	Heterogeneous graph	-	-	-	-	-	To enhance the rationality and diversity of prescriptions by combining the KG and the sequence generation model	[91]
	DGSCAM	2023	KG	106,168 “symptoms-prescription” data pairs and 25,563 “prescription efficacy” data pairs from the ancient Chinese medical books	Heterogeneous graph	37.39	25.04	29.99	-	Herbal effectiveness, herbal compatibility	Based on the Seq2Seq-based dual-branch guiding strategy, combine it with the candidate attention model to analyse the text information of symptoms	[92]
Transfer learning model	TCMBERT	2022	KG + Clinical data	1123 clinical records from a publication of a famous TCM doctor named Liu BiChen’s Experience	Heterogeneous graph	75	56	63	-	Herbal effectiveness, herbal compatibility	To reduce the model’s dependence on a large amount of labelled data by adopting transfer learning	[95]
GCN + BERT + ChatGPT	PresRecRF	2024	KG + Clinical data	14948 total samples, 1801 symptoms, 54 syndromes, and 410 herbs from the TCM-Lung database and TCM-Stroke database	Heterogeneous graph	60.24	17.74	27.41	-	-	To comprehensively integrate structured and unstructured knowledge, enhance the comprehensiveness of representation, and achieve herbal dosage prediction for the first time	[96]
Transformer-learning model + GAN	TCM prescription recommendation model	2022	Clinical data	21295 copies of EHR, covering 6352 types of medicines from Clinical record of Guang’anmen Hospital	Time series data	80.58	68.49	-	-	-	To achieve both temporal modelling ability and data augmentation effects	[97]
OSPF + ANN	TCM prescription recommendation model for COVID-19	2021	Public database	337 TCM prescriptions from TCM officially recommended in China for COVID-19	TCM database	93.20	-	85.00	-	-	To optimise prescription recommendations through the classification of medicinal properties (cold-heat attributes) and SI	[98]
GCN + BERT + GAT	BSGAM	2025	Public database	33765 processed prescriptions, 390 symptoms, and 811 types of herbs^[103]^	Heterogeneous graph	29.89	21.66	25.12	-	-	To address the issues of data sparsity, insufficient associations, and weak feature representation in traditional models	[100]
Collaborative filtering algorithm	TCMFP	2023	Public database	499 herbs, 13,144 ingredients, 785 targets, 29378 herb–ingredient relationships, and 84260 ingredient target relationships from the TCMSP database	Heterogeneous graph	-	-	-	-	-	To screen the optimal prescriptions by calculating the H - score, P - score, and FmapScore of the network after combining network science algorithms and AI	[101]
	TCM formula prediction for COVID-19	2023	Public database	6 TCM formulas, 482 TCMs, 13448 compounds, and 24 structural COVID-19 proteins from the Herbnet database of the Institute of Chinese Medicine Information, Chinese Academy of TCM	-	-	-	-	-	-	To predict TCM compound formulas effective against COVID-19 by utilising collaborative filtering algorithms and molecular docking technology	[102]
Multi-scale CNN + genetic algorithms	NDCNN	2023	Clinical data	745 clinical cases from public full text database and the RuiKang Hospital affiliated to Guangxi University of Traditional Chinese Medicine	Heterogeneous graph	-	-	-	-	-	To conduct an efficient search using evolutionary algorithms after multi-scale feature extraction	[108]

AI = artificial intelligence, ANN = artificial neural network, BSGAM = Chinese herbal medicine recommendation based on semantically enhanced self-supervised graph convolution and multi-head attention fusion model, CNN = convolutional neural network, DGSCAM = dual-branch guidance strategy combined with a candidate attention model, GAN = generative adversarial network, GAT = graph attention network, GCN = graph convolutional network, GSK3β = glycogen synthase kinase-3β, KDHR = knowledge-driven herb recommendation, KG = knowledge graph, KG-ASMGNN = knowledge graph enhanced attentive multigraph neural network, MGAT = meta-path guided graph attention network, MGCN = multigraph convolutional network, MLP = multilayer perceptron, MLR = multiple linear regression, NFFGRAM = nonlinear multifeature fusion and gated recurrent self-attention mechanism, OSPF = ontology-based side-effect prediction framework, PresRecST = TCM prescription recommendation network architecture for real-world patients with integration of syndrome differentiation and treatment planning, SI = safety index, SMRGAT = MultiGraph Residual Attention Network and Semantic Knowledge Fusion, SVM = support vector machine, SSTM = symptom term mapping method, TCM = traditional Chinese medicine, TCM-Lung = TCM Lung disease knowledge graph, TCMFP = traditional Chinese medicine formula prediction approach, TCMPR = traditional Chinese medicine prescription recommendation, TCMSP = traditional Chinese medicine systems, pharmacology database, and analysis platform.

## 6. Clinically-oriented Chinese herbal medicine recommendation model evaluation method

**Figure 4.**
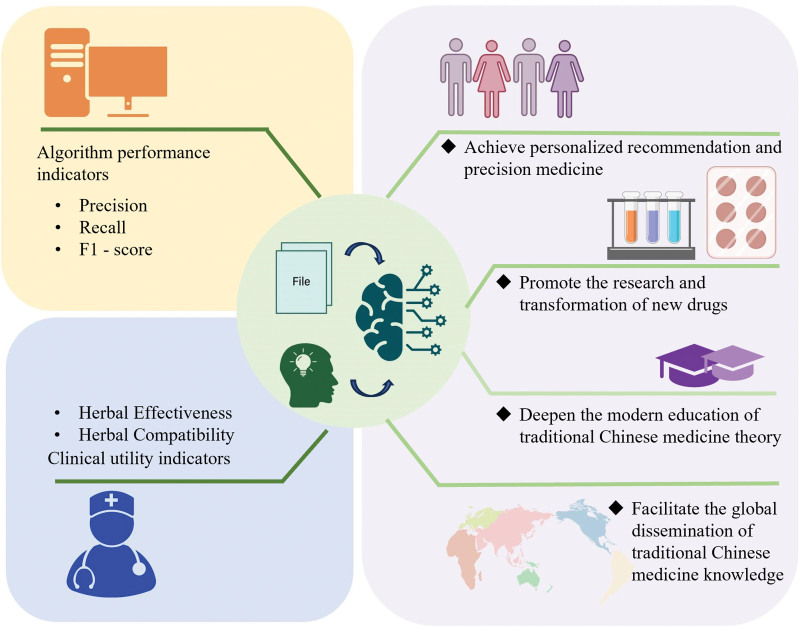
Evaluation methods and application scenarios of the Chinese herbal medicine recommendation model. Algorithm performance indicators offer an objective basis for comparing different intelligent Chinese herbal medicine recommendation models. Evaluating with unified indicators allows for intuitive assessment of each model’s pros and cons. In contrast, clinical utility indicators emphasize the model’s actual impact on patient treatment and consider practical factors that algorithm performance indicators overlook.

Reliable evaluation methods are the core guarantee for validating the performance of Chinese herbal medicine recommendation models and enhancing their clinical applicability.^[109–111]^ Currently, the commonly used evaluation methods can be divided into algorithm performance indicators and clinical utility indicators (Fig. 4). Combining the 2 can comprehensively evaluate the technical effectiveness and practical value of the model.^[112]^ Algorithm performance indicators provide an objective standard for comparing different intelligent recommendation models of Chinese herbal medicines. By evaluating with unified algorithm performance indicators, one can intuitively see the advantages and disadvantages of each model. Different from algorithm performance indicators, clinical utility indicators focus more on the actual impact of the model on patient treatment. Meanwhile, they also take into account many practical factors that algorithm performance indicators cannot cover, such as the availability of Chinese herbal medicines and patient compliance. Combining the 2 can comprehensively evaluate the technical effectiveness and practical value of the model.^[112]^

### 6.1. Automatic quantitative evaluation metrics

Algorithm performance indicators are mainly used to quantify the model’s fitting ability to labeled data. Precision reflects the proportion of effective traditional Chinese medicines in the recommended prescriptions. Recall measures the model’s ability to recall effective conventional Chinese medicines from standard prescriptions. The F1-score is the harmonic mean of Precision and Recall, which balances the contradiction between the 2.^[113–115]^ Recommendation models such as TCMPR, MGCN, and KDHR choose Precision, Recall, and F1-score as the main evaluation metrics. The TCMPR model has an accuracy of 28.23, a recall of 12.98, and an F1 score of 17.78, indicating that there is a large amount of ineffective content in its recommendations.^[71]^ However, it can serve as a basic model. After analyzing the causes of errors, the feature extraction and algorithm structure can be improved. If its performance is enhanced, it is expected to assist TCM practitioners in the preliminary screening of prescriptions. The MGCN model shows strong abilities in screening and identifying effective samples and performs well in balancing accuracy and recall.^[75]^ In the aspect of Chinese herbal medicine recommendation, it can provide accurate and comprehensive suggestions for traditional Chinese medicine practitioners, improving the efficiency and quality of prescription. It can also be applied in fields such as TCM research and development and resource management. The overall performance of the KDHR model is not good.^[77]^ Although its current application is limited, it can be improved by analyzing data quality and model structure issues. If its performance is improved, it may be used for the recommendation of TCM for specific diseases. Overall, evaluated by algorithm performance indicators, the MGCN model has good prospects, while the TCMPR and KDHR models need improvement.

For ranking-based models, it is necessary to introduce the Hit Ratio@K to evaluate whether the top K recommended results contain effective prescriptions. This indicator does not focus on the overall accuracy and comprehensiveness of the recommended results. Instead, it emphasizes whether the top-ranked recommendations can hit the valid results. In the scenario of Chinese herbal medicine recommendation, TCM practitioners may prefer to quickly see the top few prescriptions that are most likely to be effective. The Hit ratio@10 of Fordnet is 46.89%, indicating that the model can, to a certain extent, meet users’ needs for quickly obtaining valid information, but there is still significant room for improvement.^[70]^

### 6.2. Human qualitative evaluation metrics

Although traditional algorithm indicators can reflect technical performance, it is not easy to directly map them to clinical outcomes. To address this, models such as DGSCAM and TCMBERT have proposed innovative evaluation frameworks, introducing doctors’ experience to correct data biases.^[92,95,116]^ Herbal effectiveness represents clinicians’ scores (ranging from 0–5) for the main therapeutic effects of prescriptions based on traditional Chinese medicine theory. Herbal compatibility is used to evaluate traditional Chinese medicines’ synergistic or conflicting properties.

Based on symptoms related to urticaria, DGSCAM compared the similarities between the prescriptions generated by itself, those generated by the baseline model, and the label prescriptions. Meanwhile, professional doctors carried out an artificial qualitative evaluation. The results showed that the prescriptions generated by DGSCAM were roughly similar to the label prescriptions. Moreover, for secondary symptoms, DGSCAM could supplement the prescriptions with Chinese angelica root, dried rehmannia root, and dahurian angelica root, which are used for nourishing blood, activating blood circulation, and calming wind to relieve the patient’s condition, conforming to the logical thinking of traditional Chinese medicine.

TCMBERT integrates diverse TCM knowledge with deep learning for prescription generation. In a case of internal cold stagnation, it produced a comprehensive formula for diarrhea but prioritized secondary symptoms over primary manifestations (cold limbs and abdominal pain). Notably, it failed to select the aconite–cinnamon pair (standard for addressing the root pattern). Consequently, clinicians assigned a low score despite the prescription containing only 1 extraneous herb. This reveals TCMBERT’s insufficient grasp of prescription principles, particularly its inability to distinguish symptom priority and match herbs accordingly.

The above case analysis of Chinese herbal medicine recommendation can provide valuable references for the improvement and optimization of existing models. By comparing the performance of different models in actual cases, we can clearly identify their advantages and disadvantages.

## 7. Discussion

Globally, AI has become one of the core driving forces of the fourth industrial revolution.^[117,118]^ With its powerful data-processing capabilities, machine-learning algorithms, and intelligent decision-making technologies, AI has triggered disruptive changes in numerous fields. Against the backdrop of the development trend of “AI + Traditional Chinese Medicine,” the research on TCM prescription recommendation models holds significant practical importance.^[119,120]^ These models can integrate massive amounts of TCM clinical data, ancient literature data, etc, and use AI algorithms for analysis and mining. They can provide doctors with more precise and personalized prescription recommendations. This not only helps to enhance the scientific nature and accuracy of TCM clinical decision-making but also promotes the global dissemination and application of TCM, making greater contributions to solving human health problems.

Significant progress has been made in data-driven Chinese herbal medicine recommendation research. By combining KG, deep learning, and hybrid models, the research has not only overcome the limitations of traditional empirical medicine but also initially achieved the quantitative expression of TCM theories. The quantitative expression of TCM theory has been initially achieved by combining KG and deep learning models. However, both KG and deep learning have their own limitations and advantages (Table 3). KG highly relies on structured data.^[108]^ In TCM, structured data may include standardized herbal names, properties, and predefined relationships between symptoms, syndromes, and herbs. KG can clearly show the connections between symptoms and syndromes, and which herbs are commonly used to treat specific syndromes, facilitating understanding of TCM prescriptions’ internal logic for rule-based reasoning and knowledge discovery. In contrast, deep learning has a data-hungry nature.^[121]^ It needs a large amount of high-quality data to train an effective model. In TCM, collecting a large and diverse dataset covering various diseases, symptoms, and corresponding prescriptions is challenging. Insufficient data may cause overfitting in deep-learning models, that is, the model performs well on training data but poorly on new, unseen data. Models based on GCN can mine potential compatibility rules from the multidimensional relationships among symptoms, syndromes, and Chinese herbal medicines. Meanwhile, the transformer model dynamically captures the nonlinear associations between complex pathogenesis and prescriptions through the self-attention mechanism. These technological innovations provide a methodological basis for the precise recommendation of TCM prescriptions.

**Table 3:** Comparative analysis of KG and deep learning approaches for Chinese herbal medicine recommendations.

Comparison dimension	Knowledge graphs	Deep learning
Data requirements	Requires structured triples	Demands large-scale prescription datasets
Interpretability	Explicit representation of TCM theories	Implicit feature learning
Cold-start performance	Stable performance for new diseases when basic knowledge exists	Severe overfitting with insufficient data
Computational efficiency	Fast real-time inference but labor-intensive construction	High training costs but efficient prediction
TCM theory integration	Encodes explicit rules	Struggles with dynamic syndrome differentiation logic
Clinical applicability	Best for classical formula recommendations with clear rules	Effective for personalized prescriptions in complex cases
Implementation challenges	Vocabulary standardization and relationship maintenance	Data scarcity for rare syndromes and model overfitting

KG = knowledge graph, TCM = traditional Chinese medicine.

As a traditional empirical medicine, TCM adopts a combined evaluation model of computer-based evaluation and evaluation by experienced humans when assessing Chinese herbal medicine recommendation models. In human evaluation, the main indicators are herbal effectiveness and herbal compatibility, with a scoring range of (0, 5).^[122]^ Higher scores indicate better efficacy. However, human evaluation is inherently subjective, influenced by evaluators’ experience, knowledge, and theoretical perspectives. Despite this subjectivity, the combined evaluation model remains valuable. Computer-based assessment provides objective, efficient data references that mitigate bias, while human evaluation compensates for computers’ limited contextual understanding by incorporating core TCM concepts. This complementary approach yields more comprehensive and clinically relevant model selection. Accurate, effective models assist clinicians in rapidly developing personalized treatment plans, advancing TCM standardization and modernization. This evaluation paradigm bridges traditional TCM wisdom with modern technology, enhancing clinical applicability and supporting TCM inheritance and innovation.

**Figure 5.**
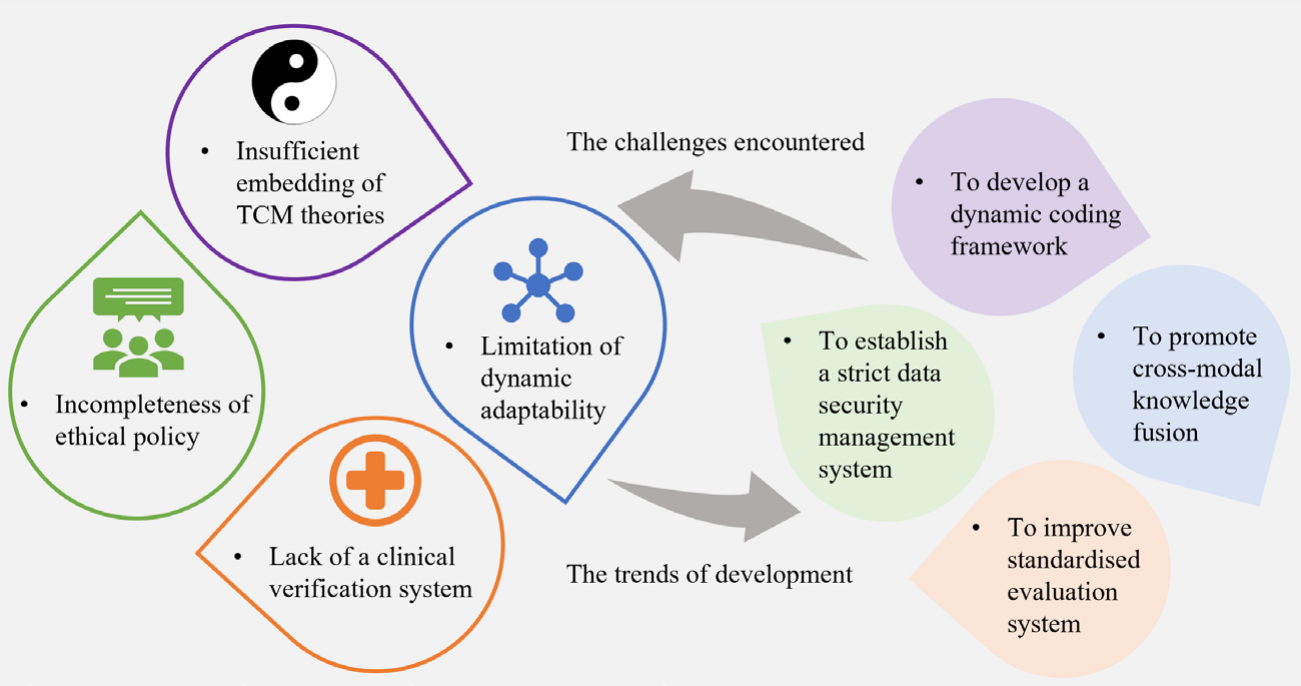
Challenges and future trends of Chinese herbal medicine recommendation models. The future development trend of Chinese herbal medicine recommendation models is to develop a dynamic coding framework, promote cross-modal knowledge fusion, improve the standardized evaluation system, and establish a strict data security management system, thereby enhancing model performance and promoting clinical application.

However, the existing research still faces 3 core challenges (Fig. 5). Firstly, there is an insufficient embedding of TCM theories. Most models still rely on rule constraints or simple weight allocation to quantify core TCM theories such as MMAG and properties, flavors, and meridian tropisms, lacking systematic mathematical modeling.^[123,124]^ The selection of the principal herb in recommended prescriptions is mostly indirectly reflected through the influence of the target network, making it difficult to simulate the decision-making process of TCM practitioners who dynamically weigh pathogenesis and constitution.^[125–127]^ Secondly, dynamic adaptability has limitations. Existing methods have weak capabilities in modeling the timing of prescriptions and cannot effectively support the clinical need of treating according to the syndrome.^[128–130]^ When a patient’s condition evolves from exterior to interior syndrome, the model has difficulty adjusting the prescription strategy in real-time (e.g., transitioning from Mahuang Decoction to Chengqi Decoction).^[129]^ Thirdly, there is a lack of a clinical verification system. Most studies rely on algorithmic indicators (such as F1-score) or manual scoring, lacking long-term efficacy tracking based on real-world data.^[131,132]^ Although a small number of studies have proposed an evaluation model that combines automatic quantitative evaluation metrics with human qualitative evaluation metrics, this model still has many problems. Specifically, it suffers from drawbacks such as a relatively single data source, overly subjective evaluation methods, and lack of specificity and over-generality in evaluation indicators. In other words, although the prescriptions generated by the model conform to theoretical logic, their clinical safety still needs to be verified through multicenter trials.

To address the above challenges, future research can make breakthroughs in the following directions: develop a dynamic coding framework for TCM theories.^[133–135]^ Transform syndrome differentiation and treatment into a causal inference graph to further quantify the impact of syndrome transformation on prescription adjustment. At the same time, use reinforcement learning to simulate the clinical thinking of TCM practitioners, especially the trial-and-error thinking of national TCM masters, to achieve personalized iteration of prescriptions. Promote cross-modal knowledge fusion. Integrate multi-omics data (metabolomics, microbiomics, metagenomics) with clinical phenotypes to analyze the multi-target synergistic mechanism of compound prescriptions.^[136–138]^ Combine metabolic pathway enrichment analysis with the manifestations of the damp-heat syndrome in TCM to reveal the bidirectional regulatory effect of the compatibility of Coptidis Rhizoma and Magnoliae Officinalis Cortex (Hou Po) on the gut microbiota-immune axis.^[139–142]^ Promote standardized evaluation and clinical translation. Establish data collection standards covering the entire process, including symptoms, signs, diagnosis results, and medication information.^[138–143]^ Adopt internationally recognized data storage formats, such as JSON or XML, to ensure the compatibility and exchangeability of data among different systems and platforms. Introduce indicators such as clinical effectiveness, safety, and economy. According to strict clinical trial designs, the effectiveness and reliability of the model in different clinical scenarios must be verified. Continuously adjust the model parameters and algorithms based on clinical feedback to promote the implementation of the model from the laboratory to the clinic.

## 8. Conclusion

Data-driven Chinese herbal medicine recommendation systems are gradually bridging the gap between empirical traditions and modern science. Their true value lies in tackling the core issues of non-computable theories and clinically unverifiable practices. Future progress demands interdisciplinary collaboration across TCM, AI, and systems biology, along with the establishment of international standards. This will drive TCM from empirical inheritance to precision computing, enabling safer, more efficient, personalized treatments for global patients.

## Acknowledgments

The authors would like to express their gratitude to Professor Yang Zhang for participating in the evaluation of the quality of the included references. They also thank Professor Hui Chen for assessing the professionalism of the discussion on TCM theories.

## Author contributions

**Conceptualization:** Meng Yang, Xin Zhou.

**Data curation:** Meng Yang, Jing Jin, Ya-Li Liu, Wei-An Hao, Xin-Yi Ao, Xin Zhou.

**Formal analysis:** Hui Chen.

**Funding acquisition:** Meng Yang, Zhi-Lin Ran, Zhi Li, Xin Zhou.

**Methodology:** Jing Jin, Xin Zhou.

**Software:** Ya-Li Liu, Long Zhao.

**Supervision:** Xin-Yi Ao.

**Validation:** Wei-An Hao.

**Visualization:** Meng Yang, Ya-Li Liu, Xin Zhou.

**Writing – original draft:** Meng Yang, Xin Zhou.

**Writing – review & editing:** Jing Jin, Zhi-Lin Ran, Zhi Li.
